# Object-based attention modulates the discrimination of level increments in stop-consonant noise bursts

**DOI:** 10.1371/journal.pone.0190956

**Published:** 2018-01-24

**Authors:** Blas Espinoza-Varas, Jeremiah Hilton, Shaoxuan Guo

**Affiliations:** 1 Department of Communication Sciences & Disorders, University of Oklahoma Health Sciences Center, Oklahoma City, OK, United States of America; 2 Department of Biostatistics & Epidemiology, University of Oklahoma Health Sciences Center, Oklahoma City, OK, United States of America; 3 College of Medicine, University of Oklahoma Health Sciences Center, Oklahoma City, OK, United States of America; Baycrest Health Sciences, CANADA

## Abstract

This study tested the hypothesis that object-based attention modulates the discrimination of level increments in stop-consonant noise bursts. With consonant-vowel-consonant (CvC) words consisting of an ≈80-dB vowel (v), a pre-vocalic (Cv) and a post-vocalic (vC) stop-consonant noise burst (≈60-dB SPL), we measured discrimination thresholds (LDTs) for level increments (ΔL) in the noise bursts presented either in CvC context or in isolation. In the 2-interval 2-alternative forced-choice task, each observation interval presented a CvC word (e.g., /pæk/ /pæk/), and normal-hearing participants had to discern ΔL in the Cv or vC burst. Based on the linguistic word labels, the auditory events of each trial were perceived as two auditory objects (**Cv**-v-**vC** and **Cv**-v-**vC)** that group together the bursts and vowels, hindering selective attention to ΔL. To discern ΔL in Cv or vC, the events must be reorganized into three auditory objects: the to-be-attended pre-vocalic (**Cv**–**Cv**) or post-vocalic burst pair (**vC**–**vC**), and the to-be-ignored vowel pair (v–v). Our results suggest that instead of being automatic this reorganization requires training, in spite of using familiar CvC words. Relative to bursts in isolation, bursts in context always produced inferior ΔL discrimination accuracy (a context effect), which depended strongly on the acoustic separation between the bursts and the vowel, being much keener for the object apart from (post-vocalic) than for the object adjoining (pre-vocalic) the vowel (a temporal-position effect). Variability in CvC dimensions that did not alter the noise-burst perceptual grouping had minor effects on discrimination accuracy. In addition to being robust and persistent, these effects are relatively general, evincing in forced-choice tasks with one or two observation intervals, with or without variability in the temporal position of ΔL, and with either fixed or roving CvC standards. The results lend support to the hypothesis.

## Introduction

In the perception of speech, the sensitivity to level differences in stop-consonant noise bursts plays an important role because such differences facilitate categorization along place of articulation [1], voicing [2, 3], and other phonetic continua. For instance, discerning between the consonant-vowel syllables /bæ/ and /pæ/ depends partly on the level of the consonant plosive noise bursts, being louder for the voiceless than the voiced consonant. Likewise, in a labeling task with a synthesized /pa-ta/ continuum, the probability of alveolar /ta/ responses increases if the noise burst level is increased relative to the vowel level [1]; that is, the noise-burst level is perceived in relation to the vowel level. Although these noise bursts are much briefer and softer than the vowel, their loudness difference is perceptually significant for consonant recognition.

Recently, the interest in studying sensitivity to consonant noise-burst level differences has increased because they can be distorted by hearing-aid processing schemes such as dynamic-range compression and enhancement of consonant-vowel intensity ratios (CVR), and the perceptual significance of these distortions becomes a question [4, 5, 6]. For instance, with 12-ms release time, fast-acting wide-dynamic range compression of vowel-consonant syllables (e.g., /ip/) increases the level of the stop-consonant noise bursts, reflected in acoustic measurements such as the envelope-difference index (EDI) and the CVR [4]. While the acoustic evidence is ample and solid, much less is known about the perceptual significance of such increments and more specifically about the differential sensitivity to increments in the burst level or the accuracy with which they are discerned [6]. Determining perceptual significance is important because, by being much briefer and lower in level than the immediately preceding or following vowels, the stop-consonant noise bursts are less audible and vulnerable to intra-speech or context interference such as temporal masking [7] that degrades the bursts perceptual analysis. Owing to this interference, the perceptual significance of burst-level differences is not equivalent to the acoustic one, and the discordance between perceptual and acoustic magnitude is not fully understood. Thus far, CVRs or EDIs have been expressed mostly in acoustic dB units, downplaying the notion of a perceptual CVR or EDI that does not depend solely on dB ratios. In consonant-vowel-consonant (CvC) words such as /pæk/, a number of contextual variables could potentially influence the perceived significance of increments in the consonant-burst level. Among these variables is whether the burst is pre- or post-vocalic, the duration of silences preceding or following the consonant-release burst, the vowel level, the uncertainty about which burst conveys a level increment, and others; however, few studies have investigated how these variables modulate the perceptual effect.

Another perceptual-significance issue relevant to CvC words is whether differential sensitivity is uniform or varies depending on which phoneme carries a level increment: the consonants, the vowel, or both. For example, to assess the effects of dynamic-range compression on sensitivity to speech-level differences, level discrimination thresholds (LDTs) for whole CvC words were measured in hearing-impaired users of compression or linear hearing aids [8]. In both amplification conditions, the level differences encompassed the full CvC word, including the noise-bursts of both consonants plus the vowel. Since there were no significant differences between the LDTs measured in the two amplification conditions, the authors concluded that the perceptual significance of compression effects was small, possibly because of the low hearing-aid compression ratios. However, this conclusion assumes constant differential sensitivity across all CvC phonemes; an alternate interpretation is that the LDTs reported in [8] are for level differences in the vowel rather than in the much briefer and softer consonant noise bursts, in which the compression effects could be stronger, but this possibility remains to be tested. Likewise, in moderate to moderately-severe hearing-impaired participants, significant recognition improvements were observed [4] for /ip/, /ik/ but not for /it/ when the consonant noise-burst level was increased by compression with 12-ms release time. However, with pre-vocalic stop consonants, the recognition improvement would not be the same as with post-vocalic stops. In vowel-consonant syllables, the noise burst of a post-vocalic stop-consonant is preceded by silence and prior to this the vowel amplitude decays to zero; in contrast, no clear-cut acoustic discontinuity is observed between the pre-vocalic stop-consonant noise bursts and the high-amplitude vowel onset. Context interference could be stronger for pre- than for post-vocalic stops.

Presently, research is needed to understand how level increments in stop-consonant noise bursts are processed at the perceptual level, and the first step is to examine how the differential sensitivity for these noise bursts relates to that for non-speech random noise used in psychoacoustic studies. This would determine whether the size of burst-level differences produced by compression is large enough relative to the resolving power of the auditory system. The next step is to study differential sensitivity for level increments in stop-consonant noise bursts embedded in CvC words, which can be viewed as an instance of level discrimination for noise targets in the presence of either a backward (for pre-vocalic bursts) or a forward (for post-vocalic bursts) vowel masker. Backward- or forward-masked level discrimination has been studied mainly for brief (e.g., 20–30 ms) pure-tone targets masked by a loud (≈ 90 dB SPL), 100-ms pure tone or narrow-band noise with 100-ms of silence between the target and masker; for recent reviews, see [9, 10, 11, 12]. A prominent effect is that, for mid-level targets (≈60 dB), the masked level-discrimination threshold (LDT) can be as much as 20 dB higher than the unmasked LDT, although the masker does not reduce the target audibility [13]. The decrease in differential sensitivity is explained on the assumption that the target and masker are integrated or grouped together at the perceptual level, (i.e., they are processed as a single auditory object) which interferes with selective attention to the target level [9]. The integration strength would increase directly with the time proximity and the similarity of the target and masker, and what confuses the observer is the task-irrelevant level fluctuation between the target and the masker taking place on each observation interval: relative to the 60-dB target, a level increment obtains with the 90-dB backward masker and a steep drop in level obtains with the 90-dB forward masker. These conditions increase the LDT because the observer has to attend to the small task-relevant increment in target level while attempting to ignore the larger but task-irrelevant level difference between the target and the masker; the target-to-masker level fluctuation comingles with the task-relevant increment in target level. Because the loudness of mid-level targets and the masker are integrated in some way, participants are unable to dissociate the loudness of the target from the masker, and this increases the LDTs by effectively reducing the signal-to-noise ratio [10]. At very low (e.g., 20 dB) or high (e.g., 100 dB) target levels, the LDT does not increase because the target and masker are not integrated or grouped together. In the aggregate, these effects point to the involvement of central perceptual-grouping rather than peripheral auditory-masking mechanisms [9].

**Fig 1:**
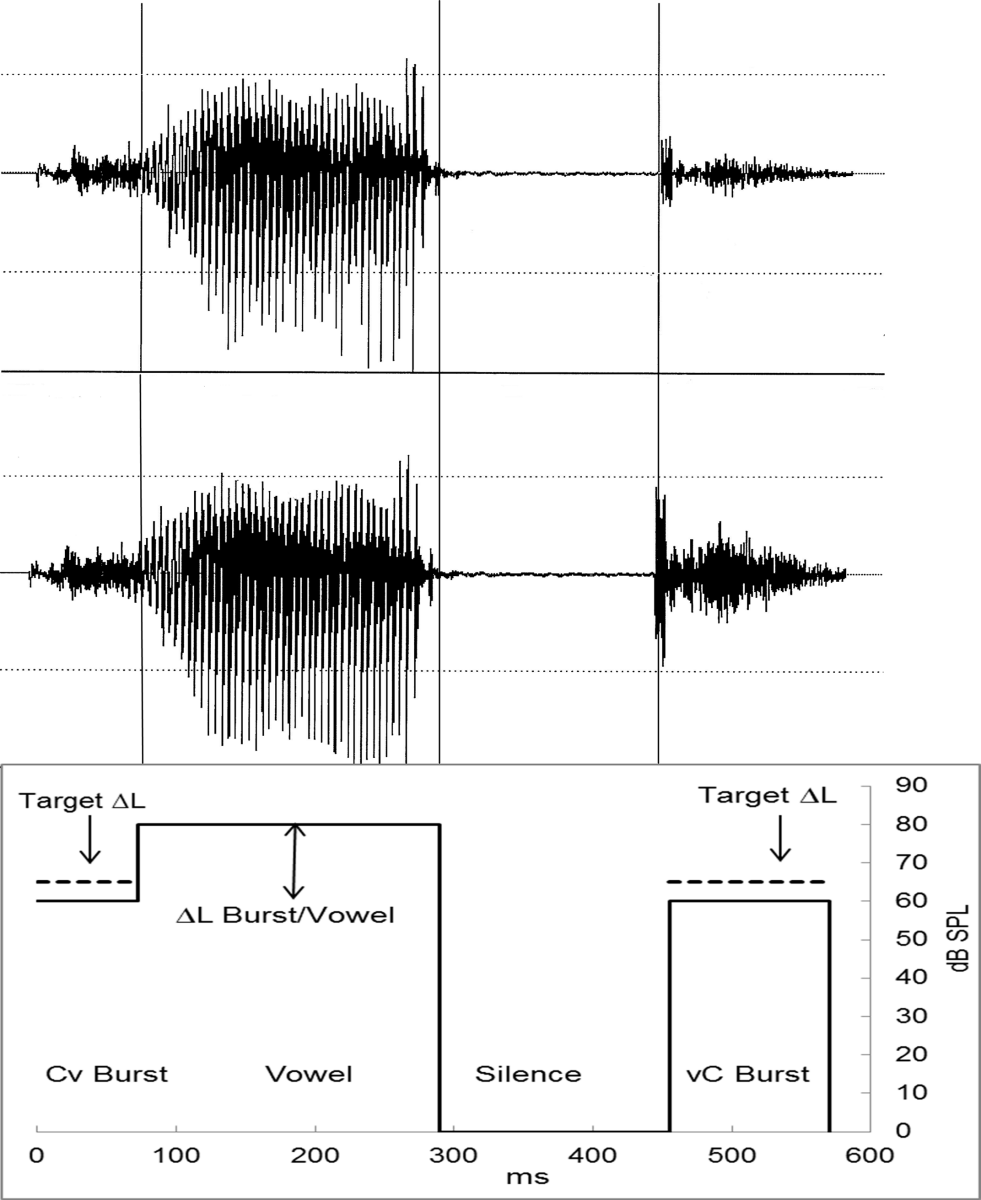
Time waveform and average amplitude-envelope of the word /pæk/. The top panel shows the original waveform, without level increments; the center panel shows the same waveform with a 6.0 dB increment in the RMS level of the post-vocalic stop-consonant burst. Vertical lines show the boundaries between four segments defined in the syllable. In the bottom panel, the abscissa shows the average duration (ms) of the four segments delimited on each CvC word: pre-vocalic burst (Cv), vowel (v), silence, and post-vocalic burst (vC). The ordinate shows the average peak dB SPL of each segment, highlighting the level relation between the noise-burst target and the vowel masker. Dashed horizontal lines depict the level increment (∆L) in the stop-consonant noise-burst target.

In the above studies, the targets and maskers were quite different from the noise-burst (targets) and vowel (masker) of the present CvC words (e.g., /pᴂk/), however the masked level-discrimination paradigm is germane to CvCs for several reasons: first, in CvC words, the pre- (Cv) and post-vocalic (vC) noise bursts and the vowel are tightly grouped or integrated together, perceived as a single auditory object (i.e., the word) and focusing attention on consonant noise bursts requires selective attention [14]. Second, in the above studies, the level disparity between the target and the masker that produced large LDT elevations is similar to the level disparity between the stop-consonant noise-burst (i.e., targets) and the /ᴂ/ vowel (i.e., masker) of CvC words: in both conditions, the target is 20–30 dB lower than the masker. For instance, in the natural CvCs of the present experiments the average level of the noise bursts and vowel was ≈60 and ≈80 dB, respectively (Fig 1 below). Third, in two-observation interval two-alternative forced-choice (2I-2AFC) tasks, discerning (across the observation intervals) a small task-relevant level difference between a standard and a comparison prevocalic noise burst also requires ignoring the within-observation interval task-irrelevant level difference between the noise burst (target) and the vowel (masker). Fourth, just like in the pure-tone studies, inclusion of task-irrelevant masker level information on the task-relevant decision variable (i.e., target level) could account for elevation of masked LDTs [10, 11, 12]. Clearly, for CvC words, discrimination of level increments (∆L) in the pre- (Cv) or post-vocalic bursts (vC) adjoining the vowel (v) can be viewed as an instance of level discrimination in conditions that hamper the perceptual segregation of the bursts from the vowel.

In order to measure LDTs for level increments in stop-consonant noise bursts, a 2I-2AFC task would present the same CvC word on each interval (e.g., /pæk/ /pæk/) and, on each trial, the full sequence of auditory events would be **Cv**-v-**vC** and **Cv**-v-**vC,** with ∆L in the Cv or vC burst (Fig 1). If attention is focused on the linguistic information (i.e., on word recognition), the auditory events comprising each word would be grouped together and perceived as a single, tightly-integrated auditory object [15]. Typically, object-based attention operates in the recognition of words and in discerning one word from another, which requires integration of many features (e.g., amplitude envelope, formant frequencies) and makes it difficult to attend selectively to details of the sounds comprising the word [9]. Discerning ∆L would require ignoring the linguistic information and attending selectively in order to reorganize the auditory events into three separate auditory objects [16]: the to-be-attended pre-vocalic (**Cv**–**Cv**) or post-vocalic burst pair (**vC**–**vC**), and the to-be-ignored vowel pair (v–v). Discrimination accuracy would depend partly on how effectively the participant can extricate the noise-burst objects from the vowel object. Previous research [17, 18] shows that, perceptually, participants are capable of separating consonant noises from words, but rather than being automatic, this process requires attention. In addition, with narrow-band noise targets followed by an uninformative noise flanker, the ability to discriminate between targets improves with increasing time separation between the target and flanker because it facilitates attending to the noise target [19]. For CvC words, this suggests that attending selectively to the stream of bursts would be much harder with pre- than post-vocalic bursts because the former are briefer and continuous with the vowel, but the latter are longer and apart from the vowel by as much as 190 ms of silence. In terms of streaming and promoting object-based attention, the post-vocalic bursts would be more effective than the pre-vocalic ones, and discriminating ∆L in noise bursts would be more accurate for post- than pre-vocalic bursts; i.e., the temporal-position effect would be strong. The ≈80-dB vowel in between the Cv and vC burst (≈60 dB each) is irrelevant to the discrimination task but is hard to ignore because it produces a 20-dB level increment (i.e., 60 to 80 dB) that can blur the perceptual magnitude of the much smaller increment in target level (∆L). The net effect of this blurring is equivalent to adding internal noise, which would be greater for the pre- than the post-vocalic burst because of the closer time proximity. However, even with post-vocalic bursts, the vowel effect would not be nullified and discrimination accuracy would not be as precise as with bursts presented in isolation, excerpted from the CvC (i.e., the vowel context effect would be significant). Lastly, varying CvC dimensions that do not alter the perceptual grouping would be expected to have minor effects on discrimination of ∆L in the noise bursts. If the study results confirm the above predictions, they would support the hypothesis that object-based attention modulates the discrimination of level increments in stop-consonant noise bursts.

Although an important effect of dynamic-range compression and of CVR enhancement is to increase the level of stop-consonant noise bursts [4, 20], little is known about the differential sensitivity for these level increments. Therefore, in this study the aim was to estimate the bursts LDTs and to determine how they depend on contextual variables; in turn, these estimates could help gauging the perceptual significance of burst-level increments stemming from compression or CVR enhancement. To characterize differential sensitivity for level increments (∆L) in the pre- (Cv) or post-vocalic (vC) stop-consonant noise bursts of CvC words, we studied how the LDTs depend on four factors. a) The presentation format of the noise bursts, whether in isolation (i.e., excerpted from the CvC) or in word context, in order to unveil context interference [7]. b) The temporal position, pre- (Cv) or post-vocalic (vC), of the burst with ∆L. c) The variability in the temporal position of the burst plus ∆L, and in the consonant producing the burst. d) The inclusion versus omission of a within-trial CvC reference that highlights the level difference between the standard and comparison CvC.

## Materials and method

### Stimuli

Speaking American English at conversational level, a 23-years-old female produced tokens of the consonant-vowel-consonant (CvC) words /pæt/, /pæk/, and /kæt/, that were digitized (20 kHz) and saved as audio files with overall duration of 574, 565, and 540 ms, respectively (mean = 560 ms). On each CvC sound file (Fig 1), four time segments were delimited. The first is the release burst and aspiration noise of the pre-vocalic stop consonant, termed “Cv” burst, lasting 65, 75, and 75 ms for /pæt/, /pæk/, and /kæt/, respectively (mean = 72 ms). The second is the vowel, “v,” lasting 152, 165, and 187 ms, respectively (mean = 168 ms), and the third is the silence preceding the final stop (mean = 190 ms). The fourth is the release burst and aspiration noise of the post-vocalic stop consonant, termed “vC” burst, lasting 167, 135, and 88 ms (mean = 130 ms). In duration, the pre-vocalic (Cv) bursts are shorter than the post-vocalic ones (vC) but, to retain real-life conditions, the natural acoustic dimensions were not altered (except for the level increments). In what follows, the noise bursts are referred to as Cv or vC, and not as phonemes (e.g., /p/) because the burst is only a fragment of this phoneme.

Hearing-aid compression alters the acoustic properties of stop-consonant noise bursts in more ways than just the level [4, 5]. Thus, in this study, level increments confined to a single consonant noise burst (Cv or vC) were implemented off-line with a digital waveform editor that increased the burst level without changing its time waveform, spectrum, or any other CvC segment [5, 21, 22]. Achieving this with a real-time programmable attenuator is impractical because real-time attenuation changes from one to another segment of a CvC word (e.g., increasing only the initial burst level) produces audible clicks and interruptions. With the waveform editor, the amplitude, no (t), of the burst sample points was multiplied by (1+c), 0 ≤ c ≤ 5.1 to produce linear increments in RMS amplitude (as well as in pressure in dyne/cm^2^) much like those of linear amplification. For example, multiplication by a factor of 2 (i.e., c = 1.0), doubles the peak-to-peak amplitude of every zero-crossing undulation in the noise waveform, resulting in a 6.0-dB increment; a factor of 1.12 produces 1.0-dB level increments. All level increments were positive to approximate the effects of compression and consonant-vowel ratio enhancement on the bursts level. In general, the RMS-level difference, ∆L in dB, between a burst without and one with positive RMS-level increments is equal to 20 log (1+c); thus, in 5–11 steps of ≈0.4–3.0 dB each, ∆L ranged from 0.21 to 14.0 dB (these are the nominal dB values computed with the equation; the acoustic precision was ≈0.5 dB). With each CvC word, a 5–11 token series was created in which the tokens differed from one another only in the size of ∆L in the Cv burst, being 0 dB (i.e., c = 0) in the standard CvC and 0.21–14.0 dB in the comparison CvC. This series and a similar one with ∆L in the vC burst were termed “in-context” because the bursts were embedded in the respective CvC word. With the words /pæk/ and /pæt/, two additional burst-in-isolation series presented only the Cv or only the vC burst, excerpted from the CvC, but the waveforms and ∆L were identical to those of the in-context series. While excising the Cv burst from the vowel, the CvC waveform was stretched along the time axis and the excision was at a sample point with zero amplitude; a new sample point was chosen if, upon play back, the burst produced audible distortions or clicks. Altogether, a total of 36 bursts-in-isolation and 52 burst-in-context sound files were used in the study.

### Task and procedure

In the 2-observation interval, 2-alternative forced choice (2I-2AFC) task of experiment 1, burst-in-context trials presented a standard CvC word (/pæk/ or /pæt/) without ∆L and a comparison CvC with a ∆L in Cv or vC, each on a separate observation interval, their order (1^st^ or 2^nd^) being random with equal probability. Inter-observation and inter-trial intervals were 0.5 s and 5.0 s, respectively. Participants pressed keys on a keyboard to report which interval included ∆L and, except for this, the two CvCs of each trial were identical; thus, the task required attending selectively to the Cv burst while attempting to ignore the remainder of the syllable, which is challenging because the CvC word is perceived as a compact whole. To recover psychometric functions relating the correct probability, P(C), to the size of ∆L, a constant-stimulus method presented randomly with equal probability each of the 9–11 tokens (/pæk/ or /pæt/) comprising the series with increasing ∆L in Cv. The same constant-stimulus 2I-2AFC method measured discrimination of ∆L in the vC burst in CvC context, and in the two burst-in-isolation conditions presenting only Cv or vC. In counterbalanced order, the four conditions (Cv or vC, each in isolation or in CvC context) were studied in 0.5–1.5 hr sessions comprising 4–9 blocks of 50–90 trials each, with 5–7 min rest in between; audiograms were collected in the 1^st^ session. On average, each of the four conditions included 720–878 trials, except for two participants that required 1260–1710 trials, and prior to collecting data a short practice period was allowed; thus the LDTs reported herein represent performance of well-trained participants. The Institutional Review Board University (University of Oklahoma Health Sciences Center) approved the testing protocol; written consent was obtained from all participants.

### Equipment and stimulus level

From the output channel of the computer D/A converter, the electrical stimulus waveforms were low-pass filtered at 8.5 kHz (Precision Filter LP 66/88), amplified (Crown D-75), attenuated, and presented monaurally over TDH-49 earphones at an overall SPL of 79–82 dB(A). Without ∆L, the bursts overall SPL was 59–62 dB(A), or ≈20 dB lower than the vowel, on average; the pre-vocalic burst of /pæt/ was only 50 dB SPL. Since they were shorter than the 300–400 ms temporal-integration time [23], the bursts ended before the ear reached full response, which could increase the LDTs; thus, moderately high presentation levels were used to make the bursts clearly audible [4]. All SPLs were measured in a 6-cm^3^ coupler and the fast, A-weighted network of a sound-level meter (Quest 155). The stimulus presentation, timing, and response recording were controlled by a Macintosh computer and LabVIEW II software (National Instruments); all listening tests took place in a sound-treated room. In all conditions, participants entered responses on a computer keyboard and feedback on a computer monitor tallied correct or error responses.

### Participants

Participants were students or staff members at the University of Oklahoma Health Sciences Center recruited by way of personal contact or flyers posted in bulletin boards. The study enrolled a total of 17 healthy participants (13 female), native speakers of English, with chronological ages ranging from 21–47 years (mean = 26), one of whom withdrew from the study before completing the experiments and the data were discarded. Among the 13 participants of experiment 1, nine had hearing thresholds levels within the normal range (≤15 dB) at the audiogram octave frequencies. The other 4 participants had mild hearing loss and, to offset its effect, the SPL of the CvC words was increased to achieve ≥ 20-dB sensation levels, keeping constant the 20-dB difference between the bursts and the vowel level. The participants in experiment 2 were four college students, 23–25 years old, with normal hearing and experience in the experimental task. For listening, an hourly fee was paid to each participant. Upon completion of the experiments, the data were summarized and analyzed (June 2016-March 2017).

## Experiment I: Noise bursts in isolation or in word context

In two participant groups, one (n = 9) listening to /pæk/ the other (n = 4) to /pæt/, experiment 1 measured LDTs in four conditions with ∆L in either the Cv or vC burst, each presented in isolation or in CvC context. In all trial blocks of a given condition, ∆L was on a single burst and participants knew which burst they had to attend to. Studied in counterbalanced order, on each condition data collection required 1–2 test sessions, each comprising 5–7 blocks of 90–110 trials each; on average, more trials were needed in burst-in-context than in bursts-in-isolation conditions.

### Results

Although they were familiar with the CvC words, in burst-in-context conditions, the participants did not exhibit best differential sensitivity to ∆L right from the outset of the listening tests. Instead, most of them needed upwards of 200–300 trials to learn to attend selectively to the burst with ∆L and a few needed many more. Because they activate object-based attention, CvC words are perceived as tightly integrated wholes rather than as a three-phoneme sequence (i.e., noise burst, vowel, noise burst), and most participants needed practice in order to discern ∆L accurately.

**Fig 2:**
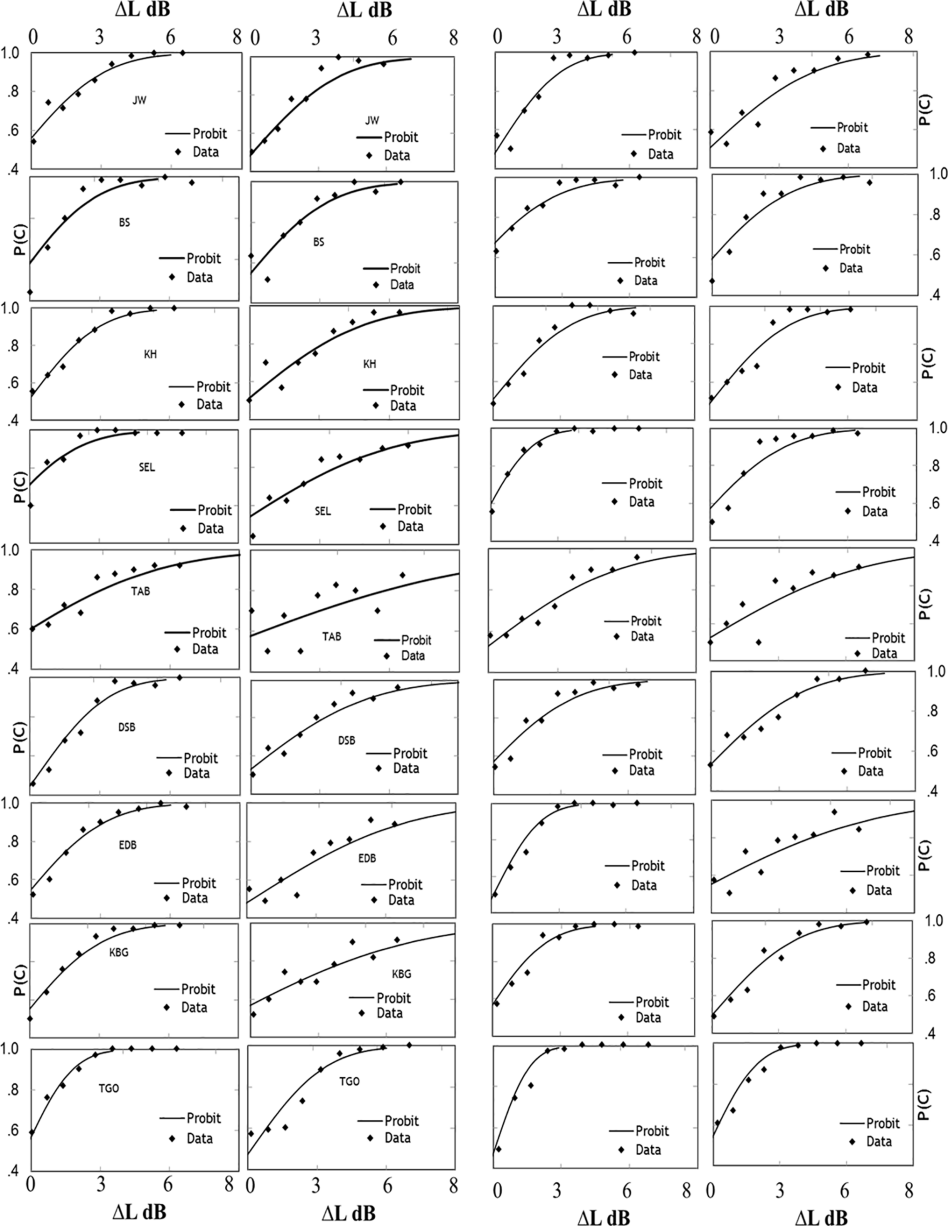
Psychometric functions relating discrimination accuracy to the burst level increment. In Fig 2, the correct probability, P(C), as a function of ∆L in dB is shown for each of 9 participants (one per row) listening to ∆L in the pre-vocalic burst of /pæk/ presented in isolation or in /pæk/ context (1^st^ & 2^nd^ column on the left-hand side). Smooth curves are maximum-likelihood probits that come closest to the data points. In the same format, the 3^rd^ and 4^th^ column on the right side show functions for ∆L in the post-vocalic burst presented in isolation or embedded in /pæk/. See Supporting Information S1 Fig for further details.

The results described below represent performance at or near the asymptotic upper limit, and include raw data from each participant since comparable evidence is not available in the open literature. Fig 2 shows representative psychometric functions for each of 9 participants tested in the four conditions with ∆L in the Cv or vC burst embedded in /pæk/ or in isolation, excerpted from /pæk/. To estimate LDTs, maximum-likelihood probits were fit to the data relating correct probability, P(C), to ∆L in dB; each data point is based on 80 trials or more. In all participants and burst conditions, accuracy increases monotonically with ∆L. For the Cv burst in isolation (Fig 2), the slopes are steep and ∆Ls ranging from 0–4.0 dB bracket the chance-to-perfect performance range. In contrast, for the Cv burst in word context, the data show more variability, the slopes are less steep, and ∆L ≥ 6.5 dB is needed to reach P(C) = 1.0; clearly, for ∆L in the Cv burst, differential sensitivity in CvC context is much lower than in isolation. Compared to those of the Cv burst, the psychometric functions for the vC burst (Fig 2B) are steeper both in isolation and in context, and the differences between these two conditions are less obvious.

**Fig 3:**
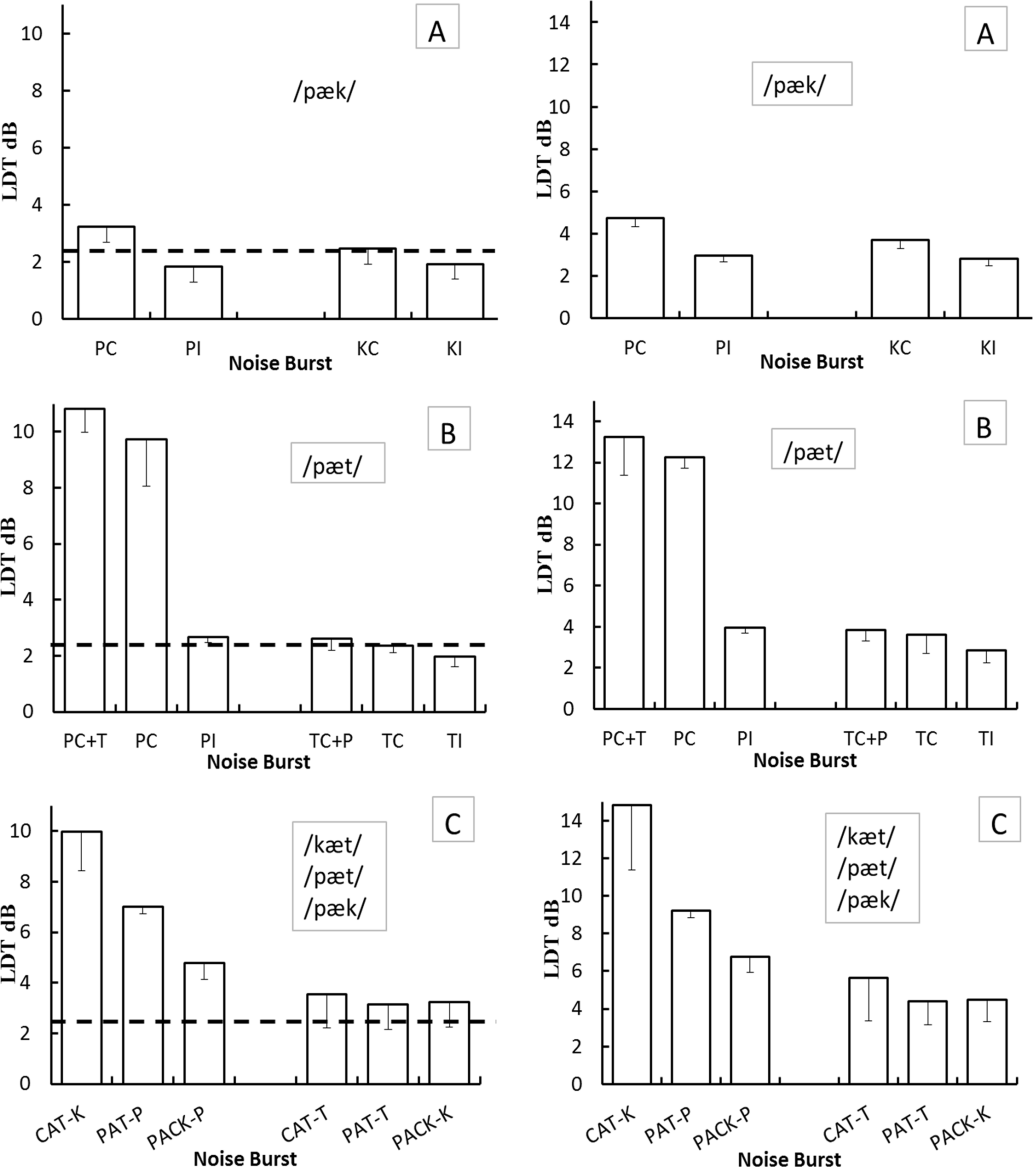
Level Discrimination Thresholds (LDTs). The LDTs (Y axis) measured in experiment 1 with the words /pæk/ and /pæt/ are depicted in the top (A) and middle panels (B), respectively; the bottom panels (C) depict the LDTs measured in experiment 2. Panels on the left- and right-hand side depict LDTs estimated at the 0.75 and 0.85 correct probability, respectively. In the top panels, for level increments in the pre-vocalic (Cv) burst of /pæk/, bars labeled PC and PI depict average LDTs (ordinate) for Cv in context and Cv in isolation; for level increments in the post-vocalic (vC) burst of /pæk/, bars labeled KC and KI depict the LDTs for vC in context and vC in isolation. For ease of comparison, on the left-hand side panels, the dashed horizontal lines depict the LDT for level increments encompassing the entire CvC word measured by [8]; since this LDT was estimated only at a correct probability of 0.71, it is not included in the right-hand side panels. In all panels, error bars depict 1.0 standard error below the mean. Middle panels (B): for level increments in the pre-vocalic (Cv) burst of the word /pæt/, bars labeled PC and PI depict average LDTs (Y axis) for Cv in context and Cv in isolation; for level increments in the post-vocalic (vC) burst, bars labeled TC and TI depict the corresponding thresholds for vC in context and vC in isolation. Bars labeled PC+T and TC+P depict LDTs measured in the word-context condition that interleaved level increments in the pre- (Cv) or post-vocalic (vC) burst within each trial block (temporal-position variability). Bottom panels (C): for level increments in the pre-vocalic (Cv) burst of /kæt/, /pæt/, or /pæk/, bars labeled CAT-K, PAT-P and PACK-P depict average LDTs (Y axis) for Cv in context; bars labeled CAT-T, PAT-T, and PACK-K depict the corresponding LDTs for level increments in the post-vocalic burst (vC). Since all six conditions were interleaved within each trial block, the task included variability in the temporal position of the level increment and in the consonant place of articulation. See Supporting Information S2 Fig for further details.

Fig 3 shows LDTs estimated from probits at P(C) = 0.75 and 0.85 (termed LDT_0.75_ and LDT_0.85_), to illustrate the consistency of the trends across two accuracy levels. The average LDT_0.75_ was 3.24, 1.84, 2.47, and 1.93 dB for Cv in context, Cv in isolation, vC in context, and vC in isolation; respectively, the average LDT_0.85_ was 4.74, 2.95, 3.71, and 2.79 dB. Repeated measures ANOVA, with burst temporal position (initial Cv versus final vC) and presentation format (in-context versus isolation) as factors revealed significant main effects of both factors and a significant interaction. With the LDT_0.75_, significant effects obtained with temporal position, F(1, 8) = 9.38, MSE = 1.036, p = .016, context-isolation, F(1, 8) = 19.45, MSE = 8.341, p = .002, and the position-context interaction, F(1, 8) = 8.17, MSE = 1.668, p = .02. With the LDT_0.85_, the position, F(1, 8) = 7.02, MSE = 3.148, p = .029, context, F(1,8) = 21.32, MSE = 16.52, p = .002, and their interaction, F(1, 8) = 5.81, MSE = 1.747, p = .042, also achieved significance. The trends are much the same at the two accuracy levels. Pairwise comparisons (2-tail Student’s t-test) of LDT_0.75_ showed significant differences between Cv context and Cv isolation (p = .001), Cv context and vC context (p = .01), and Cv context and vC isolation (p = .00); that is, the context, temporal-position, and interaction effects remain significant if the confidence level takes into account multiple comparisons (p = .017). In spite of the duration difference between the 75-ms Cv and the 135-ms vC burst, in isolation, the LDT_0.75_ did not differ significantly (p = .52); that is, the shorter duration of the Cv burst cannot account for the LDT differences observed in burst-in-context conditions.

To determine if the above trends generalize to other words, in a new participant crew (n = 4), differential sensitivity to ∆L in the stop-consonant noise bursts of /pæt/ was studied also. With the pre-vocalic burst in context, ∆L discrimination was far more difficult with /pæt/ than with /pæk/, and this enabled measuring training effects since it required many more discrimination trials. A repeated-measures ANOVA did reveal significant P(C) differences, F (1, 5) = 6.84, MSE = 1629.76, p = .047, between the initial and final set of 200 trials, each presenting a standard and a comparison /pæt/; that is, mastering the task of discerning ∆L in this condition did require significant training, even though the bursts were embedded in familiar words. Measured as with /pæk/, Fig 3 (middle panels) shows average LDTs for ∆L in the Cv or vC burst each presented in context or isolation, plus an additional subsidiary condition described below. The average LDT_0.75_ was 9.72, 2.67, 2.37, and 1.99 dB for Cv in context, Cv in isolation, vC in context, and vC in isolation; the respective average LDT_0.85_ was 12.27, 3.95, 3.63, and 2.87 dB. Repeated measures ANOVA, with burst temporal position (initial Cv versus final vC) and presentation format (in-context versus isolation) as factors, revealed significant main effects of both factors and a significant interaction. For the LDT_0.75_, significant effects obtained for temporal position, F(1, 3) = 35.13, MSE = 64.56, p = .01, context-isolation, F(1, 3) = 34.42, MSE = 55.38, p = .01, and their interaction, F(1, 3) = 18.40, MSE = 44.55, p = .023. Repeated measures ANOVA of the LDT_0.85_, showed the same significant effects: temporal position, F(1, 3) = 27.97, MSE = 94.53, p = .01, context-isolation, F(1, 3) = 64.49, MSE = 82.22, p = .04, and their interaction, F(1, 3) = 15.36, MSE = 57.19, p = .03. Pairwise comparisons (2-tail Student’s t-test) of LDT_0.75_ showed significant differences between Cv context and Cv isolation (p = .003), between Cv context and vC context (p = .014), and between Cv context and vC in isolation (p = .01). That is, the context, temporal-position, and interaction effects remain significant if the confidence level takes into account multiple comparisons (p = .017). The LDT_0.75_ difference between vC context and vC isolation was not significant (p = .16) because ∆L discrimination was very precise in both conditions. For bursts in-isolation, the difference between the average LDT_0.75_ of the 167-ms vC burst and the 65-ms Cv burst was not significant (p = .12)

Following the above, the same participants were run in a subsidiary condition that interleaved, within each trial block, the two burst-in-context conditions of /pæt/ and, at random but with equal probability, ∆L was either in the Cv or vC burst, but not in both. On a given trial, the observation interval presenting the burst with ∆L was also chosen randomly with equal probability. The goal was to determine if variability in the temporal position of ∆L would increase the LDTs since it required switching attention between the Cv and vC burst, which could hamper the ability to separate the burst auditory grouping from that of the vowel. In the subsidiary condition, testing required 8–12 blocks of 96–120 trials each, spanning 1–2 test sessions. Since all participants had practice in the preceding four conditions, this pre-training could generalize to the present condition.

With variability in the temporal position of ∆L, the average LDT_0.75_ was 10.82 and 2.61 dB for the Cv and vC burst; respectively, the average LDT_0.85_ was 13.25 and 3.83 dB (Fig 3, middle panels). A repeated measures ANOVA contrasting the LDT_0.75_ of interleaved against the non-interleaved burst-in-context condition, again revealed a significant temporal-position effect, F(1, 3) = 35.63, MSE = 201.94, p = .01, but neither the interleaving effect, F(1, 3) = 2.06, MSE = 7.20, p = .25, nor their interaction, F(1, 3) = 7.80, MSE = 4.86, p = .07, were significant. Variability in the temporal position of ∆L and switching attention across the two bursts did produce further LDT elevations but the small effects did not reach significance. This outcome is not that unexpected because this variability does not alter the perceptual grouping of the noise bursts or of the vowel, and the extensive training in the preceding, non-interleaved condition could generalize to the interleaved condition. Lack of significance could be due also to the small sample sizes, however our main goal was showing that with variability in the temporal-position ∆L participants can perform much like in no-variability conditions.

For non-speech broadband noise, 60-63-dB overall SPL, average LDT_0.71_ of 2.33 and 0.9 dB have been reported [24, 25] for 30-ms and 500-ms bursts, a duration range that encompasses the 65–187 ms of the pre- and post-vocalic (Cv and vC) consonant noise bursts used here; for ∆L in the whole CvC, the average LDT_0.71_ is 2.2 dB [8]. Compared to the sensitivity to ∆L in like-duration random noise, the average LDT_0.75_ for Cv and vC noise bursts in isolation (i.e., 1.84 & 1.93 for /pᴂk/, and 2.67 & 1.99 for /pᴂt/) fall within the LDT range for random noise and for the whole CvC; this in spite of performance-level, procedure, and stimulus differences between the previous and the present study. For both pre- and post-vocalic bursts in isolation, differential sensitivity to ∆L approaches the upper psycho-acoustic limit, implying that their duration difference could not account for the much larger temporal-position and context-effects reported here. With two CvC words (/pᴂk/ and /pᴂt/), the present results show significantly larger LDTs for bursts in word context than in isolation (a context-interference effect), for pre- than post-vocalic bursts (a temporal-position effect), and the largest LDTs for pre-vocalic bursts in context (an additive interaction of initial position and context). The temporal-position effect is much larger for /pᴂt/ than for /pᴂk/ probably because, in the former, the pre-vocalic burst (without ∆L) is ≈9.0-dB softer than the average; however, for this burst, only the in-context LDTs were enlarged, the in-isolation LDTs were not. Indexed in terms of LDTs, the perceptual significance of ∆L in noise bursts embedded in CvC words is much lower for the pre- than the post-vocalic burst; however, perceptual significance was not affected by variability in the temporal position of ∆L. The hypothesis that object-based attention modulates the discrimination of level increments in consonant noise bursts is supported by the superior discrimination accuracy observed with post-vocalic bursts, the auditory grouping of which would be more coherent than that of pre-vocalic bursts. Also consistent with object-based attention is the lower discrimination accuracy for bursts in context than in isolation, reflecting the inability to fully segregate the vowel from the noise-burst auditory grouping. Additional support accrues from the significant interaction between temporal-position and the context effect, and the similarity of the trends observed in conditions without or with variability in the temporal position of ∆L, since the latter does not alter the bursts auditory grouping.

## Experiment II. Variability in burst temporal-position and place of articulation

Since each trial block of experiment 1 presented a single CvC word, the main goal of experiment 2 was to determine if the effects observed therein would obtain also in conditions presenting a set of CvC words that brings about trial-to-trial stimulus variability. In the trial blocks of experiment 2, there was combined variability in the consonant that produced the noise burst and in the temporal position of ∆L (Cv or vC), while keeping the same vowel /ᴂ/. Each trial block presented one of three possible CvC words, /pᴂt/, /pᴂk/, or /kᴂt/, and ∆L in either the Cv or the vC burst was always in CvC context. The CvC word set included /pᴂk/, wherein /p/ is the same bilabial stop consonant of /pᴂt/, but /k/ is a velar instead of an alveolar stop consonant, /kᴂt/ wherein /k/ is a velar but /t/ is a an alveolar stop consonant, and /pᴂt/. Since all three CvCs included the same vowel /ᴂ/ bracketed by noise bursts, word variability could have only modest effects on the perceptual grouping of the noise bursts and the vowels, and would interfere only minimally with selective attention to ∆L in the noise burst. In other words, the word variability effect could be only modest because it would not affect the perceptual grouping of auditory events, which depends mainly of similarity and time proximity.

### Task, stimuli, and procedure

The main conditions of experiment 2 employed the same 2I-2AFC task of experiment 1, but within each trial block the standard and comparison were tokens of one of three CvC words (/pᴂt/, /pᴂk/, or /kᴂt/) sampled randomly but with nearly equal probability within each trial block. In addition, ∆L occurred randomly with equal probability in either the Cv or the vC burst of each word. Although larger than in experiment 1, the word set in experiment 2 remained small because the constant-stimulus method required a ∆L series of 8–9 tokens both for the Cv and the vC burst of each word, resulting in a total of 52 sound files. In addition, as many as 4600 trials were needed to obtain trial densities sufficient to compute reliable P(C) estimates with each sound file (or ∆L size). Data collection required 4–5 sessions, each consisting of 6–8 blocks of 90–110 trials.

### Results

As in experiment 1, for each participant, the LDT_0.75_ and LDT_0.85_ were estimated from maximum-likelihood probits separately for the Cv and the vC burst of each word. For the Cv burst, the average LDT_0.75_ was 9.98, 7.00, and 4.79 dB for /kᴂt/, /pᴂt/, and /pᴂk/; for the vC burst, the average LDT_0.75_ was 3.54, 3.14, and 3.25 dB, respectively (Fig 3, bottom panels). A single-factor repeated-measures ANOVA comparing average LDT_0.75_ for the Cv and vC burst showed a significant effect of temporal position for the three-word set, F(1, 2) = 11.47, MSE = 70.05, p = .01. A second repeated-measures ANOVA with word (3 levels) and temporal position (2 levels, Cv or vC) as factors revealed a significant effect of word, F(1, 2) = 13.02, MSE = 11.47, p = .02, but not of temporal position, F(1, 2) = 6.18, MSE = 70.05, p = .13; that is, for the latter factor, the LDT_0.75_ differences were significant for the word set, but not so for individual words, because of the lower trial density. Based on the 95% confidence interval, the between-word LDT_0.75_ differences were significant for the Cv but not for the vC burst; discerning ∆L in the Cv burst was far less accurate with /kᴂt/ than with /pᴂk/ [CIs = 6.94, 13.01], [3.51, 6.07]. The between-word LDT_0.85_ differences were not significant.

As mentioned above, experiment 2 did not measure LDTs for bursts in isolation; thus, the isolation-versus-context effect was assessed against the isolation LDT_0.75_ measured in experiment 1. In spite of the sample-size differences, an ANOVA comparing the average bursts-in-context LDT_0.75_ of experiment 2 to the average burst-in-isolation LDT_0.75_ of experiment 1 did reveal highly significant context-interference effects for both /pᴂk/, F(1, 22) = 21.50, MSE = 20.44, p = .00, and /pᴂt/, F(1, 12) = 9.80, MSE = 25.90, p = .01, because in experiment 2 the LDTs were 1.0–5.0 dB larger than those of experiment 1 (see Fig 1).

### Subsidiary conditions

The above participants underwent testing in two subsidiary conditions. The first was a control presenting only /pᴂt/, with variability in the temporal position of ∆L (either Cv or vC) but not in the consonant that produced the burst. In the same participants, the difference between the LDT measured in this and in the preceding condition would estimate the effect of consonant variability alone. In the /pᴂt/ only condition, the average LDT_0.75_ of 7.52 and 3.38 dB for the Cv and vC burst was not significantly different from the average LDT_0.75_ of 7.00 and 3.14 dB measured in the three-word condition, F(1, 2) = 0.332, MSE = .432, p = .623. In addition, for the condition with /pᴂt/ only, the LDT_0.75_ values were not significantly different from those measured in the temporal-position variability condition of experiment 1. A between-groups ANOVA showed a significant effect of temporal-position, F(1, 5) = 434.85, MSE = 507.32, p = .00, but not of group, F(1, 5) = 4.65, MSE = 5.43, p = .084; that is, neither the within- nor the between-group ANOVA revealed significant differences. In the aggregate, the results suggest that, for bursts in CvC context, differential sensitivity to ∆L does not decrease further by combining variability in the consonant with variability in the temporal position of ∆L; however, this conclusion is restricted to the word /pᴂt/ (the subsidiary condition did not include /pæk/, and /kæt/) and the small, single-vowel CvC set of the present condition. This outcome is within what could be expected because in order to discern ∆L, the perceptual grouping of the noise bursts and the vowel would be much the same across all three words. Further research is needed to corroborate this conclusion in a larger speech corpus and participant sample.

The second subsidiary condition assessed how burst-level discrimination depends on having the option to notice, within each 2I-2AFC trial, the contrast between the standard and the comparison bursts of the CvC word. Such within-trial contrast is not possible in a single observation-interval 2AFC task (termed SI-2AFC) in which trials present only the standard or the comparison CvC, in random order across trials; however, the perceptual segregation of the noise-burst from the vowel is still possible. The SI-2AFC task fosters attention to within-word level differences between the bursts, and reliance on memory traces of the CvC amplitude-modulation pattern (Fig 1). To discern ∆L in the noise bursts, participants can focus on the level relation between the Cv and the vC burst of each CvC; that is, on within-word level comparisons, rather than solely on standard-to-comparison burst-level differences across the observation intervals of the 2I-2AFC trial; alternatively, participants can rely on across trials burst-level comparisons. If discrimination of ∆L depends mainly on within-word comparisons and internalized CvC memory representations, allowing a single CvC observation per trial should not be much different than allowing two observations. If discrimination of ∆L were based mainly on noticing differences between the standard and comparison presented on each trial, omitting one or the other from each trial would degrade performance. To test these predictions, in the context of the word /pᴂt/, the LDT_0.75_ for ∆L in the pre- or post-vocalic burst was measured also with a SI-2AFC task presenting only the standard or only the comparison on each trial, in random order from trial to trial; SI-2AFC discrimination accuracy was compared to that of the 2I-2AFC task of the previous subsidiary condition.

#### Stimuli and procedure

The stimuli were the same tokens of the word /pᴂt/ described earlier, but the trial structure was different: in the SI-2AFC task, each trial presented either a standard or a comparison /pᴂt/ token, each with 0.5 probability but in random order across trials. On half the trials presenting the comparison CvC, the ∆L was in the pre-vocalic burst and, in the other half, it was in the post-vocalic burst; all comparison trials, presented the bursts in the context of the word /pᴂt/. Participants reported whether or not the CvC included a burst plus ∆L. Data collection required 3–4 sessions, each consisting 8–10 blocks of 90–110 trials each; many more trials were needed in the single- than in the two-interval task because the former presents the standard and comparison CvC in separate rather than in the same trial. Since the participants had considerable training in the preceding two-observation interval task, to a large extent the present condition was also a test of how readily training generalizes across conditions.

#### Results

To compare performance across the two tasks, the SI-2AFC correct probabilities, P(C), must be adjusted to rule out the possibility that inferior performance is not due simply to having only one instead of two opportunities to listen to the CvC word on each trial. In accordance with Signal Detection Theory [26], the sensitivity index (d’) measured in SI-2AFC tasks is related to the one measured in 2I-2AFC tasks as follows: **d’**_**SI-2AFC**_ **× √2 = d’**_**2I-2AFC**_. To make this prediction, each P(C) observed in the SI-2AFC task was converted to d' units, multiplied by √2 (i.e., 1.414) and the resulting d’ value was converted back to P(C) using Elliott's tables [27] in order to obtain predicted 2I-2AFC psychometric functions and LDTs. If one assumes that listening to a single (instead of two) CvC per trial has no detrimental effect on performance then, with the above prediction, differences between SI-2AFC and 2I-2IFC thresholds should not be significant. In the single-interval task, the mean observed LDT_0.75_ for the Cv and vC burst was 7.15 and 3.02 dB and the predicted LDT_0.75_ was 7.51 and 2.78 dB. Neither the LDT predicted from the SI-2AFC task nor the one measured was significantly different from the LDT observed in the 2I-2AFC task, F(1, 2) = .046, MSE = 0.283, p = .851, and F(1, 2) = 3.006, MSE = .410, p = .225. That is, differential sensitivity to ∆L was roughly the same whether or not a standard and a comparison CvC was presented on each trial; both tasks show significantly lower differential sensitivity for pre- than the post-vocalic noise bursts and for bursts in-context than in isolation (as measured in experiment 1). This suggests that to discern ∆L, participants employ similar perceptual strategies in both tasks: they can rely on the level relation between the Cv noise burst and the vowel or the one between the Cv and vC noise burst of each CvC (or observation interval) rather than on the one between the standard and comparison noise bursts (across observation intervals). With overlearned familiar words such as the present ones, well-trained participants can exhibit much the same differential sensitivity to ∆L in both single- and two-observation interval 2AFC tasks. Everyday word identification relies mostly on long-term memory standards, and does not require external standards; thus, compared to the two-interval discrimination task, the single-interval one comes closer to real-life speech recognition. The main goal of the subsidiary experiment was to show that participants are capable of discerning ∆L in a single-observation interval task that presents only the standard or the comparison CvC on each trial; that is, a task that rules out within-trial comparison of the standard and comparison CvC. Further studies employing larger participant samples are needed to determine which task produces best performance. See Supporting Information S3 Fig for further details.

## Discussion

### Context effect and perceptual grouping of noise bursts and vowel

For noise bursts embedded in CvC words, LDTs are significantly larger than those of bursts in isolation and of whole CvCs, meaning that the embedding significantly decreases the differential sensitivity, and this context interference is more pronounced for the pre- than the post-vocalic burst. The LDT enlargement observed in test conditions that maximize discrimination accuracy is likely to be greater in conditions approaching realistic speech-recognition conditions. To account for discrimination of ∆L in stop-consonant noise bursts of CvC words, the putative perceptual strategy would need to take into account the strong effects of context and of temporal position. In addition, such strategy would need to consider the weak effects of variability (in temporal position and consonant place of articulation) and of allowing only one observation interval that eliminates the benefit of contrasting the standard to the comparison CvC within each trial. From the stand-point of object-based attention [28, 29], the phoneme sound sequences (e.g., noise bursts, vowels) embedded in words (e.g., /pᴂt/) are perceived as a “unitary object” or “integrated whole” that makes it more difficult to attend selectively to individual phoneme sounds than to the full word; this is a top-down, cognitive effect that works against the breaking down of words into separate phonemes. In agreement with this view, for noise bursts in CvC context, the LDTs are significantly larger than those for whole CvCs. In addition, for bursts in CvC context, most participants needed upwards of 300 training trials (each including a standard and a comparison CvC, or 600 total) to master the discrimination task. That is, participants had to "learn" to attend selectively to the pre-vocalic noise bursts, though they were fully acquainted with the words, and could discern one from the other without errors. With consonant-vowel syllables, failure to attend selectively to specific phonetic segments has been reported previously [14]. A similar perceptual integration or grouping of the target and masker (i.e., burst and vowel) has been proposed to account for the elevation of level discrimination thresholds for pure tone targets under backward and forward masking [9, 10]. Increasing directly with the temporal proximity and similarity between the target and the masker [30], the perceptual-integration or grouping strength could account in part for the context effect; i.e., the enlarged LDTs of pre- and post-vocalic noise bursts embedded in CvC context.

### Leakage of vowel-level information in the decision variable that encodes the noise-bursts level difference

The perceptual grouping of the burst and vowel can decrease differential sensitivity owing to a confounding of information. While deciding if a noise-burst includes ∆L, the task-irrelevant 20-dB jump from the Cv noise burst to the vowel commingles with the task-relevant information that encodes the small level difference (∆L) between the standard and comparison noise burst (across observation intervals). That is, the decision is based on a combination of task-relevant noise-burst level information and task-irrelevant vowel-level information [9, 10]. For the prevocalic noise burst, this combination seems almost inevitable because the observer is attempting to discern a small task-relevant ∆L in the noise burst while having to ignore the larger ∆L from the noise-burst to the vowel (see Fig 1), which is irrelevant to the task. In other words, in the present 2AFC tasks, discerning ∆L in pre-vocalic stop-consonant bursts depends partly on the within-observation interval (or within-word) level fluctuation (≈20 dB) from the Cv noise burst to the vowel. For instance, to be noticeable, the level increment in the target burst would have to increase the combined loudness of the pre-vocalic burst and the vowel, thereby decreasing the signal-to-noise ratio of the sensory information that encodes the level increment in the noise-burst target, because the vowel information is task irrelevant. Burst level discrimination would depend partly on the level difference between the noise bursts and the vowel rather than solely on the level difference between the standard noise burst presented in one observation interval and the comparison noise burst presented in the other interval [31, 32]. This limitation in the ability to keep apart the burst from the vowel is consistent with evidence showing that the level of prevocalic bursts is perceived in relation to the vowel level [1, 3]. With stop-consonant noise bursts, differential sensitivity to ∆L was much the same whether the 2AFC task included only one or two observation intervals; this finding suggest that participants can discern the burst ∆L partly on the basis of a within-observation interval comparison of the Cv and vC level. In addition, with familiar CvC words such as the ones used here, the memory representations can be highly accurate and function as remembered standards against which the CvC stimuli can be compared [16]; however, relying on these internalized standards does not nullify the context-interference or temporal-position effects.

### Reorganizing the perceptual grouping of auditory events: Temporal-position and variability effects

As mentioned earlier, discerning ∆L in the noise bursts requires ignoring the linguistic information and attending selectively in order to reorganize the auditory events into three separate auditory objects: the to-be-attended pre-vocalic (**Cv**–**Cv**) or post-vocalic burst pair (**vC**–**vC**), and the to-be-ignored vowels (v–v). Discrimination accuracy would depend partly on how effectively the participant can set apart the noise-burst from the vowel object. The much larger LDT elevations observed with pre-vocalic than post-vocalic bursts stem from differences in the auditory streaming of the bursts and in the acoustic linkage between the bursts and the vowel (see Fig 1). Owing to the decay of vowel amplitude and the ≈190-ms silence that precedes the stop-closure release, the stream of post-vocalic bursts disassociates from the vowel to form a coherent auditory object that activates object-based attention, enabling precise ∆L discrimination. In contrast, owing to their close temporal proximity with the vowel, the stream of pre-vocalic bursts defines an ambiguous auditory object, which hinders object-based attention and ∆L discrimination. The temporal position effect provides strong support to the hypothesis that object-based attention modulates the discrimination of level increments in stop-consonant noise bursts.

In the context of the above, it is worth noting that the temporal-position effect is not merely a result of peripheral auditory masking; in other words, claiming that degraded differential ∆L sensitivity arises because the pre-vocalic burst is more susceptible to temporal masking and loudness contrast effects. With the present CvC words, it seems unlikely that the vowel could decrease the detectability of the pre-vocalic burst because this temporal backward masking is negligible when the probe approaches the 65–75 ms of pre-vocalic bursts [33, 34] presented well above detection threshold (59–62 dB SPL). Likewise, negligible decrease in detectability would be expected from forward masking of the post-vocalic burst by the vowel masker because, in addition to its long duration and high level, this burst is separated from the vowel by the ≈190-ms silence preceding the burst release. The inferior differential sensitivity for bursts in CvC context may not be explained in terms of low burst audibility stemming from temporal masking. Previous research [35] on backward and forward masking of 20-ms filtered noise bursts by synthetic-vowel maskers showed that peripheral temporal masking has little or no effect in the perception of natural stop-consonant bursts. The relatively wide bandwidth of the noise burst (1.5–7.0 kHz) ensures detectability in spite of being lower in spectrum level.

In agreement with the above perceptual strategy, variability in the temporal position of ∆L and in the burst place of articulation had minimal effects on differential sensitivity to ∆L in the noise bursts. Because it does not alter the perceptual grouping of the noise bursts, variability along one or both of these dimensions does not degrade the perceptual processes involved in this strategy. This includes the perceptual grouping of the noise-bursts and the vowels, extricating the bursts from the vowel, and the intrusion of vowel-level information in the decision process linked to the target ∆L. The time separation between the noise burst and the vowel is effective to modulate their integration, but between-trials variability in the temporal position ∆L is not. However, it is important to keep in mind that the variability in both the consonant place of articulation and in the temporal position of ∆L was quite limited: the stop-consonant set included only three CvCs all having the same vowel /ᴂ/, the ∆L could occur in only two temporal positions, and the participants had considerable task training. Making definite conclusions requires more comprehensive experiments having wider variability ranges along the above dimensions.

### Implications for dynamic-range compression

A major (but not the only) effect of dynamic range compression is to increase the level of stop consonant noise bursts. For instance, for vC syllables consisting of the vowel /i/ and a post-vocalic stop-consonant, a ≈7.0-dB increase in the consonant-vowel intensity ratio (CVR) was observed [4] with compression release times of 12- and 100-ms, and a 2.0-dB increase with 800-ms release times. In the present study with CvC words consisting of the vowel /ᴂ/ and pre- and post-vocalic stop consonants, the average LDT_0.75_ for ∆L in post-vocalic bursts ranged from 2.37–2.47 dB (see experiment 1). The present LDTs suggest that with 800-ms release time, the CVR increase would be perceptually insignificant, which is in agreement with the negligible intelligibility improvement at the 80-dB SPL presentation level. The LDTs are in agreement also with the significant intelligibility improvement observed with compression release times of 12 or 100 ms [4]. Taking into account the large methodological differences between the two studies (e.g., vowel /ᴂ/ versus /i/, pre- versus post-vocalic noise bursts, discrimination versus intelligibility performance indices), the agreement is noteworthy, suggesting that differential sensitivity for acoustic distortions stemming from compression could predict perceptual significance, and perhaps intelligibility improvement [6, 8]. However, the present differential-sensitivity estimates suggest also that this agreement is by no means general and highlight the need to consider contextual variables such as whether the stop consonant is pre- or post-vocalic. For example, even if the compression acoustic distortions were the same for pre- and post-vocalic stop-consonant noise bursts (which are not), the distortions perceptual significance would not be equal because their significance depends on temporal position. For ∆L in stop-consonant noise bursts an important contextual variable is whether the burst is followed immediately by a large increase in the vowel level (pre-vocalic) or preceded by a large drop in vowel level and silence (post-vocalic); the perceptual significance of such level increments is much lower for pre- than for post-vocalic stop consonants. Acoustic indices such as CVR or EDI do not consider the effects of these variables. Differential sensitivity is not the same across the pre- and post-vocalic consonant, not across stop consonants differing in place of articulation (bilabial versus velar).

The present results highlight the need to take into account the perceptual significance of level differences associated with dynamic-range compression or CVR enhancement. In CvC words, the perceptual CVR of pre-vocalic consonant noise bursts is much lower than the acoustic CVR; for the post-vocalic consonant noise burst, the perceptual and acoustic CVR are similar. Efforts to improve the processing of noise bursts should focus more on the pre-vocalic than in the post-vocalic stop consonants. In regard to compression, the most significant implication of the present results is in showing that differential sensitivity to ∆L in stop-consonant noise bursts of CvC words depends strongly on cognitive processing, including perceptual grouping, selective attention, and memory. This sensitivity does not depend solely on acoustic variables such as CVR or EDI or on peripheral auditory processing; deficits in the perceptual processing of these noise bursts could result partly from failure to attend rather to hear them.

### Limitations of the present study

Since this could well be the first attempt to measure discrimination thresholds for level increments in stop-consonant noise bursts, the main goal was to determine how general and robust are the main effects (i.e., temporal-position and context); thus, a relatively wide range of conditions was examined in relatively small participant samples. Also innovative is the effort to account for the results in terms of perceptual grouping and object-based attention. Overall, the outcome of the study is quite promising, but there are important limitations the most prominent of which are the relatively small samples sizes, and the small speech corpus used (CvCs only). Future studies are needed recruiting much larger samples and employing a more representative speech corpus. Exploring the possibility of using a psychophysical method that is more efficient than the constant stimuli is also called for; because this method is far too demanding in terms trials needed to obtain reliable estimates of discrimination accuracy, testing large participant samples is almost prohibitive.

## Supporting information

S1 Fig **Supporting information Figs 2A, 2B and 3 (Panels A).** The file “S1_fig.xlsx” contains the Experiment-1 data set for conditions presenting the word /pæk/ and level increments (∆L) in the Cv (“p”) or vC (“k”) burst. In this Excel file, sheet number 1, 2, 3, 4, 5, 8, 9, 10, and 11 include the data of each of 9 participants (S1-1, S1-2, S1-3, S1-4, S1-5, S1-6, S1-7, S1-8, and S9), each tested in 4 conditions: ∆L in the Cv burst (“p”) presented in word context (labeled PAKP context) or in isolation (labeled PAKP isolation), and ∆L in the vC burst (“k”) presented in word context (labeled PAKK Context) or in isolation (labeled PAKK Isolation). For each condition, the data are shown on a different quadrant of each sheet, but the location (top or bottom, left or right) varies across participants (or sheets) so the above labels (e.g., PAKP context) should be used to find the data of each condition. On each of the four conditions (or quadrants) included on each sheet, the panel labeled “confidence limits” is the output of the probit regression comprising four columns that list, from left to right, the predicted correct probability, the predicted RMS multiplication factor (Estimate) or ∆L size needed to achieve the respective correct probability (for details, see “Stimuli” section in the manuscript), and the upper and lower bound of the 95% confidence interval. For example, in the upper left-hand quadrant of sheet 1 (PAKP Context), a 1.439 estimate is associated with a predicted correct probability of 0.8. Immediately to the right of the “confidence limits” panel, the four data columns (J, K, L and M) show the “Estimate,” the predicted correct probability (Probit), the observed correct probability (measured), and the actual multiplication factor applied to the burst waveform (e.g., 1.439, 0.8, 1.0, 1.9, respectively in row 12). The data in these four columns create the top chart under “PAKP context” in sheet 6 (column F, G, H, rows 7–13). Because the probit regression algorithm computes an estimate for each 0.05 increment in correct probability, in the 0.45 to 0.99 correct-probability range, the probit regression produces 19 (factor) estimates that were aligned with the 9 multiplication factors applied to the waveform sample points (column L, rows 6–14). In order to align them, the measured data are plotted in relation to the chart primary vertical and horizontal axes, and the predictions are plotted in relation to the chart secondary vertical and horizontal axes. Reading from the “confidence limits” panel (sheet 1, PAKP context), the discrimination thresholds estimated at the 0.75 and 0.85 correct probabilities are 1.355 and 1.536 (sheet 1, column F). In Sheet 7, these threshold values are entered under participant S1-1 (column B, rows 4–5), which correspond to increments of 2.638 and 3.727 in dB units entered in column P, rows 4–5 (to express in dB, the formula is 20 * log 1.355 and 20 * log 1.536). The same computations were performed for all four quadrants of sheets 1, 2, 3, 4, 5, 8, 9, 10, and 11, and the full set of 36 psychometric functions is charted in sheet 6 and in the manuscript Fig 2. The 0.75 (or 75%) and 0.85 (or 85%) correct-probability thresholds are summarized in sheet 7 in terms of RMS multiplication factors (columns B to J, rows 4–5, 7–8, 10–11, and 13–14) and in terms of dB (columns P to X, rows 4–5, 7–8, 10–11, and 13–14). Analyses of variance were performed on the set of thresholds (in dB units) measured in the 9-participant sample. In sheet 7, columns Y, Z and AA (rows 4–5, 7–8, 10–11, and 13–14) list the mean, standard deviation, and standard error of the thresholds in dB, and they are charted right below the data sets and in the manuscript Fig 3 (panels A).
(XLSX)

S2 Fig **Supporting information Fig 3 (Panels B).** The file “S2_fig.xlsx” summarizes the level discrimination thresholds (LDTs) measured in Experiment 1 for conditions presenting the word /pæt/ and level increments (∆L) in the Cv (“p”) or vC (“t”) burst; LDTs were estimated with the same probit-regression procedure used in the conditions presenting the word /pæk/. In the left-hand side of Sheet 1 (columns B-F), the LDTs are expressed in terms of RMS multiplication factors, and in the right-hand side (columns L-R), the LDTs are expressed in terms of dB units (i.e., 20 * log factor). For participants S2-1, S2-2, S2-3, and S2-4 (columns B, C, D, and E) and the group mean (column F), the 75% (or 0.75) and 85% (or 0.85) percent correct LDTs measured in 6 conditions are listed in separate rows as follows: p-context (rows 4 and 5), p-isolation (rows 7 and 8), t-context (rows 10 and 11), t-isolation (rows 13 and 14), p interleaved with t in context (PAT-P, rows 16 and 17), and t interleaved with p in context (PAT-T, rows 19 and 20). In the right-hand side of sheet 1, the above LDTs are expressed in dB units, for S2-1, S2-2, S2-3 and S2-4 (columns L, M, N, and O). The group mean, standard deviation, and standard error of the mean are listed in columns P, Q, and R, and charted right below the LDT values and in Fig 3, panels B of the manuscript. The LDTs in dB units (arranged in the rows as described above) were used in the ANOVAs.
(XLSX)

S3 Fig **Supporting information Fig 3 (Panels C).** The file “S3_fig.xlsx” summarizes the level discrimination thresholds (LDTs) measured in Experiment 2 for conditions presenting any of three words within each trial block, /pæk/, /pæt/ or /kæt/, sampled randomly with equal probability. In all cases, the level increments (∆L) were in the Cv or vC burst embedded in the respective words. Thresholds were estimated with the same probit regression procedure used in conditions presenting the word /pæk/. LDTs estimated at the 75, or 85% correct level (or 0.75 or 0.85 correct probability) are expressed in terms of RMS multiplication factors in columns B, C, D, and E for the word /pæt/, columns G, H, I and J for the word /cæt/ and in columns L, M, N, and O for the word /pæk/. Expressed in terms of dB units, the respective LDTs are shown in columns T, U, V, and W for /pæt/, columns Y, Z, AA, and AB for /kæt/ and columns AD, AE, AF, and AG for /pæk/. Rows 17, 18, 19 show the LDTs measured in three participants (S3-1, S3-2 and S3-3), and rows 20, 21, and 22 show the corresponding mean, standard deviation, and standard error of the mean; these LDTs are charted below the threshold values in sheet 1, and in Fig 3, panel C of the manuscript. Rows 5, 6, and 7 show LDTs for the subsidiary condition presenting only the word /pæt/, and rows 8, 9, and 10 show the corresponding mean, standard deviation, and standard error of the mean. Rows 29, 30, 31 show the LDTs measured in the subsidiary condition with a single-observation interval task, and rows 33, 34, and 35 show the LDTs predicted by Signal Detection Theory (see Experiment II, Subsidiary conditions, for details). The LDTs in columns Z and AA, rows 25 to 33 were used to assess the significance of the temporal-position effect (Cv versus vC) for all three words combined. All ANOVAs were performed on the LDTs in dB units.
(XLSX)
